# Relative sensitivity of cortisol indices to psychosocial and physical health factors

**DOI:** 10.1371/journal.pone.0213513

**Published:** 2019-04-03

**Authors:** Jerrald L. Rector, Louis Tay, Christopher W. Wiese, Elliot M. Friedman

**Affiliations:** 1 Department of Human Development and Family Studies, Purdue University, West Lafayette, IN, United States of America; 2 Department of Psychological Sciences, Purdue University, West Lafayette, IN, United States of America; 3 School of Psychology, Georgia Institute of Technology, Atlanta, GA, United States of America; Istituto Superiore Di Sanita, ITALY

## Abstract

**Objective:**

Regulation of cortisol under resting conditions is widely used to assess physical and psychological status, but due to the diversity of possible assessments (e.g., cumulative levels; diurnal patterns), considering one or a few at a time hampers understanding and interpretation. Moreover, most studies of cortisol regulation focus on negatively-valanced experiences. This study examined the inter-correlations among cortisol indices and their relative contribution to the explained variance in diverse psychosocial and health factors, including positive functioning.

**Methods:**

Data are from midlife and older adults (N = 513; 47.2% male). Cortisol was assessed in urine (overnight) and saliva (at rest and over 4 consecutive days). Positive and negative psychosocial and health factors were assessed by self-report. In addition to examining associations among cortisol indices, relative weight analysis was used to determine which indices were most robustly linked to specific psychosocial factors.

**Results:**

Inter-correlations among indices were weak-to-moderate, suggesting that they measure different aspects of hypothalamic-pituitary-axis activity. Overall variance in psychosocial and health factors (*R*^2^) explained by the cortisol indices ranged from 0.01 to 0.07. Of this explained variance, relative weight analysis showed that waking cortisol contributed most to the variance in hedonic well-being (32.1%–38.2%), bedtime cortisol to depression-related factors (32.1%–46.9%), the cortisol awakening response to eudaimonic well-being (35.8%–50.5%), cortisol slope to perceived stress (29.2%), and urinary cortisol to physical factors (38.5% and 62.7%).

**Conclusions:**

Positive and negative factors were related to largely non-overlapping cortisol indices. This study illuminates nuanced associations among cortisol indices and diverse aspects of mental and physical health, facilitating thoughtful examination of the complex role of hypothalamic-pituitary-axis activity in health.

## Introduction

Resting and non-stress cortisol levels have been assessed in different tissues (e.g., saliva, urine, blood) and over different time scales (e.g., momentary, change across the day, accumulation over many hours) [1]. While not all assessments are thought to tap into the same aspects of hypothalamic-pituitary-adrenal (HPA) regulation (e.g., the cortisol awakening response (CAR) is distinct from other diurnal cortisol measures; [2]), the extent to which they converge or diverge from one another is unclear because few studies include multiple cortisol indices within the same individuals. Similarly, few studies have examined multiple cortisol indices simultaneously to determine which among them is most robustly associated with specific aspects of mental or physical health. Thus, it is also unclear whether these indices have overlapping or distinct relationships with psychosocial factors, and further, if unique associations differ in magnitude relative to one another. Finally, the majority studies examining links between HPA function and psychosocial factors have focused on negatively-valenced experiences, such as distress and depression [3–5], leaving open the question of potential links to positive psychosocial functioning [6–8].

This study addresses these issues by examining associations among multiple cortisol indices and the relative strengths of their association with a range of both negative and positive psychosocial factors and markers of health within the same sample. Data is from a large, nationally representative sample of middle aged and older adults–the Survey of Midlife Development in the United States (MIDUS)–that includes cortisol indices reflecting cumulative (e.g., urinary free cortisol), resting, and diurnal (e.g. saliva samples at multiple times across consecutive days) aspects of cortisol regulation. MIDUS also assesses multiple aspects of psychosocial functioning (e.g., hedonic and eudaimonic well-being; depressive symptoms; perceived stress) that have been widely used in epidemiological and experimental studies. Finally, an established statistical approach–relative weight analysis [9]–was utilized to determine the strength of association between individual cortisol indices and psychosocial and health-related factors.

Due to minimal invasiveness and relative ease of collection in naturalistic settings, urinary and salivary assessments of cortisol under resting or non-stress conditions are frequently collected in epidemiological studies [10, 11] with timescales ranging from diurnal patterns across multiple days to cumulative output over shorter periods of time (typically 12–24 hours) to single time points (reviewed in [1]). Agreement among these different indices, determined in a piecemeal fashion across studies and populations, is generally weak [11, 12], suggesting these indices may reflect different features of HPA functioning. For example, the mechanisms governing the CAR and cortisol output over the rest of the day appear to be distinct [2, 13]. Although numerous studies examining predictors of diurnal cortisol curves have considered several indices at once, to our knowledge no study to date has examined the relationships among a large, diverse set of commonly used cortisol indices in a single sample. One aim of the current study was thus to determine the extent to which diverse assessments of cortisol under resting and non-stress conditions were correlated or divergent.

A second aim was to illuminate potentially nuanced associations between multiple cortisol indices and a large set of physical and mental health measures. Depression, for example, has been linked to flatter slopes of cortisol decline across the day [14] and higher awakening levels [15]. But because few studies have examined multiple indices within the same individuals, it is not clear whether depression is most robustly linked to diurnal declines in cortisol, to awakening levels, or to other aspects of cortisol regulation entirely. For similar reasons, it is not clear whether the cortisol indices that are sensitive to symptoms of depression are similarly sensitive to other aspects of mental or physical health. The current study applies relative weight analysis to multiple cortisol indices and measures of mental and physical health to illuminate the common or discrete associations among these assessments.

Finally, relatively little research has examined the links between cortisol and positive psychosocial factors [3–5, 14, 16]. This gap presents an opportunity for a broader integrative concept of health that includes the presence of positive psychosocial states and not merely the absence of negative states [8]. While dysregulated cortisol patterns have robust links with poorer psychosocial functioning [5, 17, 18], lower total cortisol output and steeper daily slopes are also associated with greater life satisfaction [19], positive affect [16], and eudaimonic well-being (purposeful engagement with meaningful life pursuits) [20]. The CAR and measures of diurnal rhythm have been linked to potentially adaptive efforts to cope with anticipated challenges of the day ahead, as well as increased psychosocial resources (e.g., social support), general health, and well-being [14, 21, 22]. The few studies aimed at disentangling the biological correlates of positive and negative psychosocial factors suggest that associations observed between positive factors and cortisol represent distinct relationships, rather than mirror images of their negative counterparts [23, 24]. This study is designed to add to the existing literature by expanding the number and types of cortisol indices and the diversity of positive and negative aspects of psychosocial functioning assessed within the same individuals.

Based on prior literature, there were limited *a priori* predictions, including strong correspondence between the daily cortisol slope and greater perceived stress [25], bedtime cortisol and depression [26], and the CAR and stress [1]. Beyond these, however, neither existing literature nor theory provided compelling guidance for specific predictions. This study was designed to extend previous lines of research by pitting a significantly broader set of seven commonly used cortisol indices (from urine and saliva) against one another using relative weight analysis, making it possible to discern the unique contribution of each individual cortisol index to the total explained variance in each negative and positive psychosocial factor, as well as metrics of physical health, within a large national sample.

## Methods

### Participants

Participants (N = 513, 47.2% male) were from the second wave of MIDUS (MIDUS 2) collected from 2004 to 2006. MIDUS comprises a national probability sample of non-institutionalized English-speaking adults living in the co-terminus United States. A detailed description of the study sample and longitudinal retention in MIDUS has been published elsewhere [27]. Collection and analysis of data were approved by the Institutional Review Boards at the University of Wisconsin–Madison and Purdue University, were conducted in compliance with the Declaration of Helsinki, and all participants provided informed consent. The current study included participants with full information on multiple measures of cortisol and key outcomes, including psychosocial factors, chronic medical conditions, and socio-demographic factors. Details of the analytic sample derivation are provided in S1 File and S1 Fig.

Information on socio-demographics, chronic conditions, psychological well-being, and life satisfaction was obtained from telephone interviews and self-administered questionnaires. A sub-sample of respondents (Diary sample) participated in a week-long study of daily experiences that included salivary cortisol collected four times a day for four consecutive days. Some respondents participated in a 2-day laboratory study (Biomarker sample) that included collection of saliva and 12-hour overnight urine samples for cortisol assessment. The latter participants also completed the Mood and Anxiety Symptom Questionnaire (MASQ), the Center for Epidemiological Studies Depression Inventory (CES-D), and the Perceived Stress Scale (PSS). The median (IQR) time elapsed between the Main and Diary samples, the Main and Biomarker samples, and the Diary and Biomarker samples was 20 months (30.5 months), 21 months (23 months), and 11 months (10 months), respectively.

### Cortisol measures

Table 1 describes each of the cortisol indices used in the current study, including the sub-sample and specimen from which it was obtained, the time frame captured, the method for its calculation (if applicable), and the biological process that it reflects. Saliva samples for diurnal cortisol patterns were collected at waking, 30 minutes later (wake +30), before lunch, and before bed. Participants were instructed to collect samples before eating, drinking, or brushing their teeth and to avoid any caffeinated products before collecting samples. Timing of samples was obtained from daily telephone interviews and paper-pencil logs. From these data, average waking and bedtime cortisol levels, cortisol awakening response (CAR: wake +30—waking), cortisol slope across the day (wake to bedtime), and area under the curve relative to ground (AUC_g_) for each participant were calculated. The reader should note that only two time points were available to assess the CAR, contrary to the three suggested in the recent expert consensus guidelines [28]. The AUC_g_ was calculated using the trapezoidal rule. Average waking and bedtime cortisol measures were calculated for those with measurements for at least 3 out of the 4 total days. Resting cortisol was a baseline sample collected in the Biomarker sample between 7:28 AM and 10:56 AM (median = 8:24 AM; IQR = 46.2 minutes) with 86.5% of the samples collected between 7:30 AM and 9:30 AM. The 12-hour overnight urine sample was collected between 7 PM and 7 AM. Some samples were excluded from the analyses–see S2 File for exclusion criteria.

**Table 1:** Description of cortisol indices used in the current study.

Index	Sub-sample	Specimen	Type	Time frame	Description
**Waking cortisol**	Diary	Saliva	Single time point	Morning	Cortisol immediately upon waking
**Area under the curve relative to ground (AUC**_**g**_**)**	Diary	Saliva	Calculation: Trapezoidal rule– 4 cortisol values (waking, waking +30 minutes, lunch, and bedtime) against collection times	Diurnal	Aggregate cortisol secretion over the course of the day
**Bedtime cortisol**	Diary	Saliva	Single time point	Evening	Cortisol at the end of the day when levels approach a nadir
**Resting cortisol**	Biomarker	Saliva	Single time point	Morning	Random, non-fasting cortisol collected between 7:30 AM and 11:00 AM
**Urinary cortisol**	Biomarker	Urine	Cumulative	12 hours overnight	Overall cortisol output during a 12-hour overnight period
**Cortisol awakening response (CAR)**	Diary	Saliva	Calculation: Difference between waking +30 and waking samples	30–45 minutes	Rapid rise in cortisol immediately after waking
**Cortisol slope**	Diary	Saliva	Calculation: Linear regression of waking, lunch, and bedtime samples (waking +30 not included) against collection times	Diurnal	Diurnal rhythm of cortisol levels typically peaking within the first hour of awakening and declining across the day to a nighttime nadir (excludes waking +30)

### Cortisol analyses

Salivary cortisol samples were collected on cotton swabs in pre-labeled salivettes (Sarstedt, Germany). All samples were frozen and shipped for analysis. At the time of assay, samples were thawed, centrifuged, and quantified using a commercially available luminescence immunoassay (IBL International, Hamburg, Germany). The reported intra-assay and inter-assay coefficients of variation were below 5%.

Urine cortisol assays were performed at the Mayo Medical Laboratory in Rochester, MN. Enzymatic colorimetric assay and liquid chromatography-tandem mass spectrometry (LC-MS/MS) was used for urine free, random, cortisol/cortisone assay. Urinary cortisol was adjusted for creatinine. The reported inter-assay coefficients of variation for urine cortisol and creatinine were 6.1% and 0.9%, respectively.

### Psychological well-being

Consistent with current perspectives on positive psychological; functioning, we assessed the HPA associations with two domains of psychological well-being: eudaimonic and hedonic. Eudaimonic well-being reflects the Aristotelian ideal of the sustained engagement with meaningful life pursuits [29, 30]. Hedonic well-being is associated with pleasure, contentment, and the avoidance of physical and mental discomfort and is typically assessed using life satisfaction and ratings of positive and negative mood [29, 31]. Eudaimonic and hedonic well-being are considered related–scores on relevant scales are typically correlated within individuals–but distinct [29, 30], with both types of well-being making contributions to diverse aspects of health and physiological functioning [32].

#### Eudaimonic well-being

Eudaimonic well-being was assessed using Ryff’s Psychological Well-Being (PWB) scales [33, 34]. These consist of six sub-scales: autonomy (Cronbach’s α = .71), environmental mastery (α = .78), personal growth (α = .75), positive relations with others (α = .78), purpose in life (α = .70), and self-acceptance (α = .84) [34]. A 7-item version of each subscale was used, and participants responded on a 7-point Likert scale (1 = ‘Strongly agree’ to 7 = ‘Strongly disagree’). Subscales were averaged to compute a composite psychological well-being score (α = .88).

#### Hedonic well-being

Life satisfaction. Participants were asked to rate their satisfaction with life overall, using an 11-point scale ranging from 0 = ‘worst possible’ to 10 = ‘best possible’.

Positive and negative affect. Symptoms of depression and anxiety were measured using the MASQ [35]. This questionnaire consists of five subscales: anxious arousal (α = .81); loss of interest (α = .82); high positive affect (α = .93); general depressive distress symptoms (α = .90); and general anxious distress symptoms (α = .82) that are aggregated to indices of anxiety, depression, and positive affect. For each subscale, participants were asked about experiences in the prior week (e.g., “Felt nothing was very enjoyable,” “Hands were shaky,” “Felt really happy”). Participants responded using a 5-point Likert scale (1 = “Not at all” to 5 = “Extremely”).

### Perceived stress scale

Perceived stress was measured by the 10-item version of the Perceived Stress Scale (PSS; [36, 37]). Participants were asked in reference to the last month how often, for example, they felt “angered because of things that were outside of your control?” Each item was rated on a 5-point Likert scale (1 = “Never” to 5 = “Very often”). Higher total scores indicated greater perceived stress (α = .86).

### CES-D depression inventory

Depressive symptoms were also assessed using the Center for Epidemiological Studies Depression Inventory (CES-D; [38, 39]). Participants were asked in relation to the past week how often they felt, for example, “fearful”, “sad” or “lonely”. These items were rated on a 4-point Likert scale from 1 = “Rarely” to 4 = “Most of the time”. Higher values indicated more depressive symptoms (α = .89).

### Chronic conditions

To examine the relative associations between cortisol and both chronic illness [40] and obesity [41, 42], numbers of chronic conditions was determined from a list of 13 conditions–heart problems, cancer, hypertension, high cholesterol, asthma, arthritis, HIV or AIDS, diabetes, tuberculosis, neurological disorders, stroke, and ulcers. Body mass index (BMI) was determined from participant-measured height and weight and dichotomized into a variable indicating obesity (BMI ≥30).

### Socio-demographic factors

Participants’ age, sex, educational attainment, and difficulty paying bills were obtained from self-administered questionnaires.

### Statistical analyses

With the exception of the 3-point slope across the day, cortisol measures were either log_10_- or square root-transformed prior to analyses to approximate a normal distribution. Analyses were performed using IBM SPSS statistics version 24 (IBM-SPSS, Chicago, IL). Two sets of zero-order correlations assessed (1) the relationship among the cortisol measures and (2) the association of the cortisol measures with socio-demographic, psychosocial, and physical health factors. Relative weight analysis was used to examine the relative importance of each individual cortisol measures in explaining the variance in the key outcome variables [43]. Compared to multivariate regression analysis, relative weight analysis helps address multicollinearity problems when independent variables are correlated with one another and partitions the explained variance (*R*^2^) among multiple predictors to examine the contribution of each predictor in the regression equation [44]. The resulting percentages, therefore, represent the relative proportion of the total *R*^2^ accounted for by each individual cortisol index. For example, if 3 independent variables (*x*, *y*, and *z*) together produce a total *R*^*2*^ of 0.12, then a relative weight of 50% for *x* would mean it uniquely contributes 0.06 to the *R*^*2*^. Supplemental analyses included variables to account for time elapsed between the Main, Biomarker, and Diary samples as well as differences in the timing of resting cortisol samples.

## Results

Table 2 shows descriptive statistics for the outcome variables and their zero-order correlations with the cortisol measures. Participants were on average 55 years old and 47% male. The average level of education was less than a 4-year degree. On average, participants reported that paying their bills was not very difficult, and most were overweight.

**Table 2:** Participant characteristics, psychosocial and physical health factors, and their correlations with the cortisol indices (N = 513).

	Mean (SD)/%	Waking cortisol	Bedtime cortisol	Cortisol awakening response	Cortisol slope	Urinary cortisol	Resting cortisol	AUC_g_^d^
**Age (years)**	54.77 (11.48)	.104*	.188***	.157***	-.055	.092*	.187***	.210***
**Sex (% male)**	47.20% (–)	-.189**	-.134**	.115**	.175***	.104*	-.175***	-.186***
**Education**	7.82 (2.47)	.089*	-.082	-.083	-.077	-.010	.019	-.033
**Difficulty paying bills**	1.85 (0.85)	-.080	-.002	-.078	.098*	-.035	.001	-.108*
**Body mass index (kg/m**^**2**^**)**	27.93 (5.51)	-.135**	.031	.070	.146***	-.142***	-.035	.007
**PWB**^**a**^ **composite score**	39.84 (5.49)	.065	-.014	.108*	-.059	.043	.029	.097*
**Autonomy**	37.64 (6.87)	.009	.051	.144***	.006	-.030	.076	.140**
**Environmental mastery**	39.61 (7.31)	.046	.006	.049	-.030	.063	.024	.080
**Personal growth**	39.90 (6.65)	.048	-.027	.080	-.044	.038	-.008	.031
**Positive relations w/ others**	41.93 (6.53)	.074	.001	.085	-.060	.041	.035	.072
**Purpose in Life**	40.35 (6.27)	.019	-.099*	.029	-.050	.042	-.011	-.015
**Self-acceptance**	39.59 (7.88)	.106*	-.008	.117**	-.096*	.047	.020	.134**
**Life satisfaction**	8.10 (1.27)	.139**	.025	.052	-.118**	.056	.001	.125**
**MASQ**^**b**^**: Depressive symptoms**	18.13 (6.12)	-.055	.092*	-.033	.092*	-.027	.006	-.043
**MASQ**^**b**^**: Anxious symptoms**	16.31 (4.24)	-.080	-.032	-.057	.062	-.052	-.030	-.101*
**MASQ**^**b**^**: Loss of interest**	11.67 (3.80)	-.075	.086	-.042	.092*	-.067	.025	-.049
**MASQ**^**b**^**: Anxious arousal**	21.35 (4.52)	-.149***	.074	-.032	.125**	-.142***	-.013	-.095*
**MASQ**^**b**^**: High positive affect**	45.54 (9.67)	.089*	-.014	.035	-.080	.041	-.010	.046
**Perceived stress scale**	21.15 (6.12)	-.055	.033	-.034	.068	-.022	-.011	-.024
**CES-D**^**c**^ **depression**	7.06 (7.32)	-.098*	.091*	-.021	.101*	-.069	.033	-.032
**Chronic conditions**	1.59 (1.49)	-.074	.072	.065	.085	-.206***	.038	.063

Coefficients significant at

*p < .05

**p < .01 &

***p < .001.

^a^Psychological well-being

^b^Mood and Anxiety Symptoms Questionnaire

^c^Center for Epidemiological Studies Depression Inventory

^d^Area under the curve relative to ground.

With the exception of the association between age and the cortisol slope, all of the cortisol indices were significantly correlated with age and sex. MASQ anxious arousal showed inverse associations with the cumulative cortisol indices (urinary cortisol and AUC_g_) and with waking cortisol, and a positive association with the cortisol slope. There was a similar pattern of associations with BMI, except the AUC_g_ association was not significant. The psychological well-being composite score, autonomy and self-acceptance showed significant positive associations with the CAR and AUC_g_. Additionally, self-acceptance, life satisfaction, and CES-D depression showed significant associations with waking cortisol and the slope across the day. Cortisol levels before bedtime was positively associated with MASQ depressive symptoms and CES-D depression scores, and inversely associated with purpose in life.

Table 3 shows the mean values of and zero-order correlations between the 7 cortisol indices. All of the correlations were statistically significant, with the exception of three relationships: urinary cortisol and bedtime cortisol, urinary cortisol and the CAR, and the CAR and resting salivary cortisol. The absolute value of the significant coefficients ranged from .088 to .864, with the majority (14/18) of associations smaller than ±.400, indicating weak-to-moderate correlations among the measures. Waking cortisol levels, the cortisol slope, and the AUC_g_ were correlated with all the other measures.

**Table 3:** Means of and zero-order correlations between cortisol indices (N = 513).

	Mean (SD)	Waking	Bedtime	CAR^a^	Slope	Urinary	Resting
**Waking (nmol/L)**	14.84 (6.14)	1	—	—	—	—	—
**Bedtime (nmol/L)**	2.74 (2.65)	.277***	1	—	—	—	—
**CAR**^**a**^ **(nmol/L)**	6.73 (7.24)	-.220***	.088*	1	—	—	—
**Slope**	-0.73 (0.35)	-.864***	.098*	.293***	1	—	—
**Urinary (μg/g)**	12.02 (2.14)	.129**	.005	.042	-.088*	1	—
**Resting (nmol/L)**	9.77 (1.78)	.171***	.164***	.004	-.128**	.115**	1
**AUC**_**g**_^**b**^ **(arbitrary units)**	132.91 (50.96)	.559***	.559***	.458***	-.316***	.105*	.196***

Significant at

*p < .05

**p < .01, &

***p < .001.

^a^Cortisol awakening response.

^b^Area under the curve relative to ground.

Table 4 shows the results of the relative weight analyses, presented as the percentage of total variance for each psychosocial or physical health measure accounted for (*R*^2^) by each cortisol index (e.g., waking cortisol) in relation to other correlated indices (e.g., slope across the day). Cortisol indices overall accounted for 1–7% of the variance in the psychosocial and physical health factors (*R*^2^ range: 0.01–0.07). These effect sizes are consistent with prior work [14, 42]. Relative weight analyses showed that within this total variance accounted for, waking cortisol contributed the highest proportion to the explained variance for education (35.1% of total *R*^*2*^), life satisfaction (32.1% of total *R*^*2*^), positive affect (38.3% of total *R*^*2*^), and anxious arousal (28.8% of total *R*^*2*^) and was the second or third largest contributor to the explained variance for many of the other factors. Bedtime cortisol was the largest contributor to the explained variance in the three depression-related factors–MASQ depressive symptoms (47.0% of total *R*^*2*^), MASQ loss of interest (37.1% of total *R*^*2*^), and CES-D depression scale (32.1% of total *R*^*2*^)–as well as purpose in life (54.0% of total *R*^*2*^). It was also the second largest contributor to the explained variance in age (25.4% of total *R*^*2*^) and education (27.1% of total *R*^*2*^). The CAR accounted for the largest proportion of the explained variance for the PWB composite score (46.1% of total *R*^*2*^) and the autonomy (35.8% of total *R*^*2*^), personal growth (50.5% of total *R*^*2*^), positive relationships with others (45.1% of total *R*^*2*^), and self-acceptance (36.2% of total *R*^*2*^) sub-scales.

**Table 4:** Percentage of variance explained by cortisol measures from relative weight analysis (N = 513).

	Age	Sex^b^	Education	BMI^c^	Chronic conditions	Perceived Stress Scale	Difficulty paying bills
**Waking**	(3.84)	7.33	**35.06**	**(23.69)**	**(10.09)**	**(23.71)**	**(18.54)**
**Bedtime**	**25.43**	(7.22)	**(27.09)**	(2.27)	4.68	10.62	6.76
**CAR**^**a**^	**21.73**	**19.78**	(10.00)	(3.01)	(2.20)	**(26.61)**	**(23.35)**
**Slope**	(5.31)	10.49	**15.55**	**22.95**	(5.87)	**29.16**	14.49
**Urinary**	5.74	11.77	(0.99)	**(38.45)**	**(62.74)**	(2.58)	(7.20)
**Resting**	**26.01**	**(17.87)**	1.69	(1.36)	3.41	(0.77)	4.48
**AUC**_**g**_^**d**^	(11.93)	**(25.55)**	(9.61)	8.27	**11.01**	6.54	**(25.18)**
Total R^2^	0.10	0.13	0.04	0.05	0.07	0.01	0.02
	**PWB**^**e**^ **composite**	**Autonomy**	**Personal growth**	**Relationships w/others**	**Self-acceptance**	**Purpose in life**	**Environmental mastery**
**Waking**	**13.23**	(5.35)	**18.77**	**22.90**	**18.41**	7.28	10.47
**Bedtime**	(7.88)	(4.40)	(4.58)	(1.91)	(7.03)	**(53.99)**	(8.74)
**CAR**^**a**^	**46.10**	**35.78**	**50.45**	**45.11**	**36.23**	**11.66**	**(11.93)**
**Slope**	9.12	(2.30)	**10.87**	**13.40**	13.59	8.62	3.89
**Urinary**	4.39	(4.72)	5.48	4.72	3.11	**9.27**	**26.33**
**Resting**	1.91	**13.39**	(0.83)	3.53	(0.37)	(0.86)	2.18
**AUC**_**g**_^**d**^	**17.37**	**34.06**	(9.02)	(8.42)	**21.27**	(8.32)	**36.46**
Total R^2^	0.02	0.04	0.02	0.02	0.04	0.02	0.01
	**Depressive Symptoms**^**f**^	**Loss of interest**^**f**^	**CES-D**^**g**^ **Depression**	**Life satisfaction**	**Positive affect**^**f**^	**Anxious arousal**^**f**^	**Anxious symptoms**^**f**^
**Waking**	10.41	**(15.19)**	**(27.44)**	**32.12**	**38.27**	**(27.91)**	**(18.87)**
**Bedtime**	**46.91**	**37.09**	**32.05**	(3.16)	(4.17)	**21.95**	3.99
**CAR**^**a**^	(6.01)	(8.25)	(4.43)	12.81	**18.48**	(3.01)	**(20.06)**
**Slope**	**18.71**	(11.94)	**(13.55)**	**23.21**	**22.86**	(11.89)	13.10
**Urinary**	(1.73)	**(12.33)**	(10.23)	6.94	6.98	**(22.92)**	(13.15)
**Resting**	0.76	3.72	4.55	(1.40)	(2.21)	0.40	(2.23)
**AUC**_**g**_^**d**^	**(15.46)**	(11.50)	7.76	**20.36**	(7.03)	(11.91)	**(28.59)**
Total R^2^	0.02	0.03	0.04	0.03	0.02	0.07	0.01

Bold values are the highest three percentages. Parentheses indicate negative regression coefficients.

^a^Cortisol awakening response.

^b^Female = 1.

^c^Body mass index.

^d^Area under the curve relative to ground.

^e^Psychological well-being.

^f^Mood and Anxiety Symptoms Questionnaire subscale.

^g^Center for Epidemiological Studies Depression Inventory.

Cortisol slope across the day accounted for the highest percentage of the explained variance for perceived stress only (29.2% of total *R*^*2*^). Daily slope accounted for the second highest proportion for MASQ depressive symptoms (18.7% of total *R*^*2*^), life satisfaction (23.2% of total *R*^*2*^), and MASQ positive affect (22.9% of total *R*^*2*^). The AUC_g_ accounted for the highest proportion of the explained variance for sex (25.6% of total *R*^*2*^), environmental mastery (36.5% of total *R*^*2*^), difficulty paying bills (25.2% of total *R*^*2*^), and MASQ anxious symptoms (28.6% of total *R*^*2*^), and contributed the second highest proportion to chronic conditions (11.0% of total *R*^*2*^), the PWB composite (17.4% of total *R*^*2*^), autonomy (34.1% of total *R*^*2*^), and self-acceptance (21.3% of total *R*^*2*^).

Urinary cortisol was the largest contributor to the explained variance of the physical health-related factors–chronic conditions (62.7% of total *R*^*2*^) and BMI (38.5% of total *R*^*2*^)–and accounted for the second or third highest proportion of the explained variance for purpose in life (9.3% of total *R*^*2*^), environmental mastery (26.3% of total *R*^*2*^), MASQ loss of interest (12.3% of total *R*^*2*^), and MASQ anxious arousal (22.9% of total *R*^*2*^). Resting cortisol was the highest contributor to the explained variance for age (26.0% of total *R*^*2*^) only.

Supplemental analyses, adjusting for the time intervals between samples showed nearly identical results. Additionally adjusting for the collection time of the resting sample, the top two contributors remained unchanged with a few notable exceptions–resting cortisol became the second highest contributor for purpose in life (30.7% of total *R*^*2*^), personal growth (17.0% of total *R*^*2*^), and MASQ anxious symptoms (19.3% of total *R*^*2*^) and was no longer the top contributor for age (1.6% of total *R*^*2*^).

## Discussion

The current study examined the relationships among commonly used cortisol indices under resting and non-stress conditions, as well as the relative importance of these indices in explaining the variance of positive and negative psychosocial and physical health factors. The results showed largely weak-to-moderate correlations among indices, adding to prior evidence that different assessments of HPA function under non-stress conditions are separate and have distinct interpretations [2, 10–13]. In the same vein, relative weight analysis showed different cortisol indices were related to measures of mental and physical health in distinct ways. Below, these results are examined in the context of the broader literature, highlighting some potential implications.

Associations among different cortisol indices have typically been compared piecemeal across studies in different populations, meaning the degree to which the most common indices relate to one another has remained unclear. Nonetheless, the relatively weak correlations among diverse cortisol measures observed in the current study were of similar magnitudes to those reported previously, including indices of single time points, short-term collections (24 hours or less), and diurnal patterns across days [11, 12, 18, 45]. The present results using a broad set of cortisol indices in a large national sample thus bolster existing research suggesting that diverse measures of cortisol under resting and non-stress conditions tend not to be strongly related. Elucidating the reasons for this general lack of agreement among cortisol indices across arguably similar contexts (e.g., the lack of systematic differences in marked stressors) is a promising agenda for future research. Highly focused research, for example, has yielded detailed characterization of the CAR [2, 13, 17]. A similar strategy could be applied to other dimensions of cortisol functioning under resting and non-stress conditions to illuminate the factors that influence the degree to which they diverge from one another.

Relative weight analysis revealed nuanced patterns between specific cortisol indices and clusters of related factors. Broadly, positive psychosocial functioning was most strongly associated with morning cortisol assessments while negative functioning was most strongly linked to late-day assessments and declines across the day.

The CAR and waking cortisol levels reflect processes involved in the transition from sleep to wakefulness [2, 13]. Specifically, the CAR is thought to facilitate a physiological and psychological “boost” of resources in response to the anticipated demands of the day ahead [46, 47]. In the current study, waking cortisol levels and the CAR explained the largest proportion of the variance in the positive psychosocial factors (hedonic and eudaimonic well-being). Waking cortisol also explained substantial variance in markers of better overall health (e.g., lower BMI, fewer chronic conditions, lower levels of stress). Collectively, these results suggest that higher waking cortisol levels and larger amplitude CAR may be linked to well-being broadly construed.

In contrast, explained variance in measures of depression and perceived stress were most substantially contributed by bedtime cortisol levels and by declines in cortisol across the day, respectively. Bedtime cortisol represents HPA activity just prior to sleep as levels decline toward the nocturnal nadir. Elevated evening cortisol levels have been found in depressed patients [26] and may be linked to abnormal circadian rhythmicity [48]. Daily declines in cortisol reflect both starting (i.e. morning) and ending (i.e. bedtime) values. Daily cortisol slope contributed to a substantial proportion of the explained variance in perceived stress in the current study, an observation that is consistent with stress-related flattening of diurnal cortisol rhythms, including lower morning cortisol values and higher afternoon and evening values, reported in a meta-analysis [25]. Thus, the present results suggest that while positive aspects of psychosocial functioning are linked to HPA function related to the beginning of a typical day, negative functioning may be more strongly associated with the extent to which the HPA activation is able to wane optimally in preparation for sleep.

Urinary cortisol reflects longer-term (often 12-hour) accumulation of cortisol in the body, and in this study it was the top contributor to the explained variance in the number of chronic conditions and BMI. Specifically, the strong contribution (63% of total *R*^*2*^) of lower urinary cortisol may more generally represent a link between chronic conditions and hypocortisolism. This possibility is supported by previous research in which hypocortisolism was linked to higher BMI and was observed in patients with chronic conditions like arthritis and asthma [41, 49, 50], although the reasons for lower cortisol levels in the context of some aspects of poorer health are unclear.

Cortisol indices that coincided strongly with positive factors did not tend to similarly coincide with negative factors, suggesting that positive psychosocial functioning may have unique associations with features of HPA activity and are not the merely the mirror image of negative factors [16, 23]. Similar differential associations have been observed in studies examining broader biological correlates of positive and negative psychosocial factors [20]. Interestingly, there was also some heterogeneity in the relationships observed among positive psychosocial factors themselves. For example, the CAR was the greatest contributor to the variance in most of the eudaimonic well-being factors, whereas waking cortisol accounted for the most variance in hedonic well-being factors. Hedonic and eudaimonic domains of well-being are argued to be related but distinct aspects of positive psychological functioning [30, 51]. An earlier study in a sample of aging women distinguishing between these domains of well-being likewise found differential associations with various biological correlates, including the cortisol slope [17]. Thus, the nuanced associations between HPA function and psychosocial processes may include not only differences between the positive and the negative, but also between different formulations of positive well-being.

Some features of the current study limit the conclusions about the associations between HPA functioning and psychosocial experience. First, an average of diurnal cortisol indices across the four days of cortisol sampling was used. Associations between day-to-day variability in cortisol regulation and psychosocial factors were beyond the scope of this study. Nonetheless, it is worth noting that up to 50% of the variance in diurnal cortisol indices can be attributed to day-to-day fluctuations [52], making the relationship between variation in daily cortisol patterns and diverse psychosocial processes an important target for future research [46, 53]. Second, the median time between the Dairy and Biomarker data collection was 11 months, a time gap that could have contributed to the weak correlations between the some of the cortisol indices. To address this possibility we conducted additional analyses adjusting for differences in the timing of sample collection and observed similar associations (see S1 Table). Moreover, indices collected within the same sub-study where cortisol assessments were synchronous in time were also weakly correlated. Finally, all of the correlations among cortisol indices observed here were of similar magnitude to those reported in prior studies, suggesting that the differences in the timing of data collection likely did not affect the magnitude of what appear to be stable associations among cortisol measures under resting conditions.

Third, as this study dealt primarily with resting and non-stress samples, these results may not generalize to situations when stress levels are elevated and positive and negative affect are proposed to show an inverse relationship [54]. Finally, the proportion of variance explained for each physical or mental health factor was low (*R*^2^ ranging from 0.01 to 0.07). However, it is important to note here that the *R*^2^ values observed here are consistent with typical associations between any biological and psychosocial factor (e.g., [14, 23]) and small effect sizes do not necessarily indicate small practical effects. Psychosocial and physical health are highly multi-faceted, and while cortisol may be an integral contributor, there are many other factors, including genetics and environment, that contribute to the variation in the factors measured in this study [55].

In spite of these limitations, the current study has notable strengths, including a large and diverse sample of community-dwelling adults, diverse assessments of resting and non-stress cortisol in different contexts, a rich array of measures of psychosocial functioning and health, and an analytical approach designed to illuminate the strengths of association between different cortisol indices and psychosocial and health measures. Previous studies, including those using the cortisol data from the MIDUS cohort, have contributed well-developed models that frame our understanding of how certain cortisol indices are related to many psychosocial factors, including socioeconomic status, life satisfaction [19], well-being [23], depression [56], negative affect [57], daily stressors, positive events [7, 58, 59], and physical health [60]. This study adds to this prior work by explicating the extent to which cortisol indices used across these studies represent the same or distinct underlying aspects of HPA axis activity and the degree to which each is linked to diverse psychosocial and health factors. Overall, the current study provides a rigorous examination of nuanced associations among these measures and underscores the complexities inherent in linking HPA regulation to psychosocial experience.

## Supporting information

S1 Fig Derivation of the analytical sample. MIDUS–Survey of Midlife Development in the United States; P2 –Project 2 of MIDUS 2; P4 –Project 4 of MIDUS 2.
(TIF)

S1 File Analysis sample derivation. (DOCX)

S2 File Cortisol data exclusion criteria. (DOCX)

S1 Table Supplementary relative weight analyses adjusted for time intervals (N = 513). Original–unadjusted; Sample–adjusted for time between Main, Diary, and Biomarker samples; Sample/Rest–additional adjustment for time of resting sample collection. Bold values are the top three contributors. Negative values represent negative regression coefficients.
(DOCX)
