# Supplementary figures and images for: Relative sensitivity of cortisol indices to psychosocial and physical health factors
Source: PLoS One. 2019 Apr 3;14(4):e0213513. doi: 10.1371/journal.pone.0213513 (PMC6447160; doi:10.1371/journal.pone.0213513)

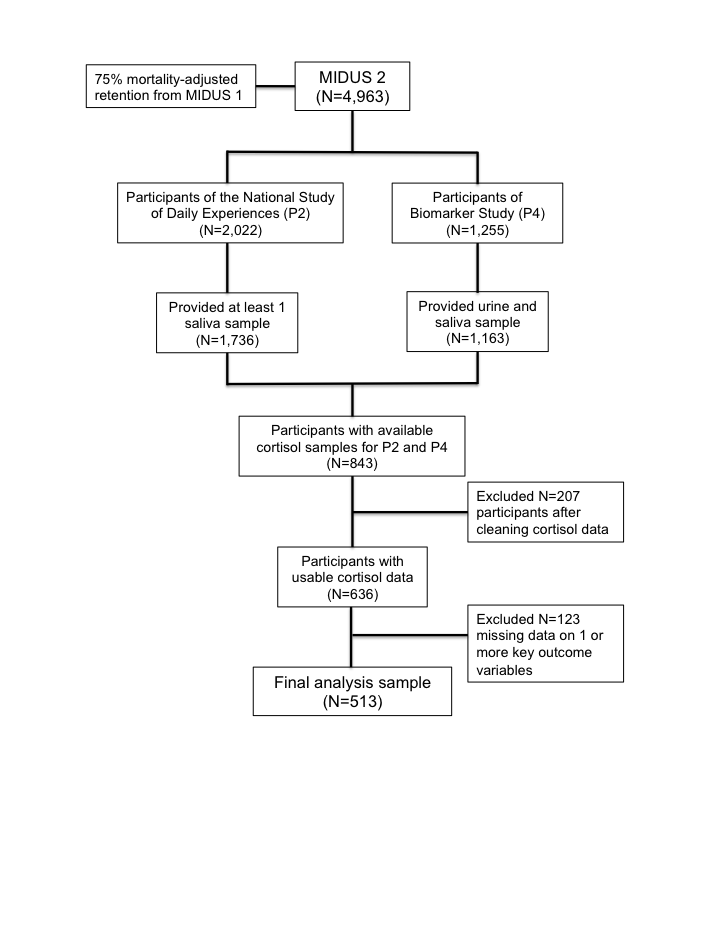

Supplement: S1 Fig — MIDUS–Survey of Midlife Development in the United States; P2 –Project 2 of MIDUS 2; P4 –Project 4 of MIDUS 2. (TIF) [file pone.0213513.s001.tif]
