# Supplementary material for: Relative sensitivity of cortisol indices to psychosocial and physical health factors
Source: PLoS One. 2019 Apr 3;14(4):e0213513. doi: 10.1371/journal.pone.0213513 (PMC6447160; doi:10.1371/journal.pone.0213513)
Supplement: S1 File — (DOCX) [file pone.0213513.s002.docx]

# Analysis sample derivation

S1 Fig. shows the sample derivation for the current study. Briefly, N=1,736 (85.9%) of the Diary study participants provided salivary cortisol samples and N=1,163 (92.7%) of the Biomarker study participants provided urine and saliva samples. There were N=843 participants with salivary and urinary cortisol data from both the Diary and Biomarker sub-studies. A further N=207 participants were excluded during the data cleaning procedures described below, leaving N=636 participants with usable cortisol data. Finally, those missing data for one or more psychosocial, health, or socio-demographic measures were excluded from analyses (N=123), leaving N=513 participants with full information (Figure 1). Age and sex of the analytical sample were not different from the full MIDUS 2 sample, although the analytic sample had better overall self-reported mental and physical health, were more likely to be White, to have higher education, and to have less difficulty paying their bills.
