# Supplementary material for: Relative sensitivity of cortisol indices to psychosocial and physical health factors
Source: PLoS One. 2019 Apr 3;14(4):e0213513. doi: 10.1371/journal.pone.0213513 (PMC6447160; doi:10.1371/journal.pone.0213513)
Supplement: S2 File — (DOCX) [file pone.0213513.s003.docx]

# Cortisol data exclusion criteria

Diary sample data exclusion criteria included: (a) samples in which raw cortisol values were >60 nmol/L, (b) days in which participants woke up before 4 A.M. or after 12 noon, (c) days in which the participant was awake for <12 h or >20 h, and (d) days in which participants’ lunch levels were ≥10 nmol/L higher than their wake +30 samples, as this is an indicator of non-compliance. The CAR was excluded if the wake +30 sample was provided either <15 min or >60 min after waking, potentially missing the CAR. Biomarker sample data were excluded if (a) salivary cortisol values were >100 nmol/L for any sampling point (n=3) or (b) the urinary cortisol collection period was >13 hours or <11 hours (n=10).
