# Supplementary material for: Relative sensitivity of cortisol indices to psychosocial and physical health factors
Source: PLoS One. 2019 Apr 3;14(4):e0213513. doi: 10.1371/journal.pone.0213513 (PMC6447160; doi:10.1371/journal.pone.0213513)
Supplement: S1 Table — Original–unadjusted; Sample–adjusted for time between Main, Diary, and Biomarker samples; Sample/Rest–additional adjustment for time of resting sample collection. Bold values are the top three contributors. Negative values represent negative regression coefficients. (DOCX) [file pone.0213513.s004.docx]

Table S1: Supplementary relative weight analyses adjusted for time intervals (N=513)

|  | **Age** |  |  | **PWB composite** | |  | **MASQ depressive symptoms** | | |
| --- | --- | --- | --- | --- | --- | --- | --- | --- | --- |
|  | Original | Sample | Sample/Rest | Original | Sample | Sample/Rest | Original | Sample | Sample/Rest |
| Waking | -3.84 | 3.73 | 5.04 | **13.23** | **13.54** | **13.51** | 10.41 | 9.98 | 9.23 |
| Bedtime | **25.43** | **29.53** | **38.57** | -7.88 | 8.61 | 8.09 | **46.91** | **47.88** | **44.28** |
| CAR | **21.73** | **19.69** | **23.03** | **46.1** | **45.44** | **42.41** | -6.01 | 5.80 | 5.47 |
| Slope | -5.31 | 5.43 | 7.49 | 9.12 | 9.02 | 8.92 | **18.71** | **18.57** | **16.50** |
| Urinary | 5.74 | 7.18 | 9.57 | 4.39 | 3.61 | 2.81 | -1.73 | 1.72 | 1.00 |
| Resting | **26.01** | **23.28** | 1.63 | 1.91 | 2.18 | 8.10 | 0.76 | 0.66 | 10.31 |
| AUC_g_ | -11.93 | 11.15 | **14.66** | **17.37** | **17.60** | **16.16** | **-15.46** | **15.40** | **13.22** |
| Total R^2^ | 0.10 | 0.09 | 0.08 | 0.02 | 0.03 | 0.03 | 0.02 | 0.02 | 0.03 |
|  | **Sex** |  |  | **Autonomy** |  |  | **MASQ loss of interest** | |  |
|  | Original | Sample | Sample/Rest | Original | Sample | Sample/Rest | Original | Sample | Sample/Rest |
| Waking | 7.33 | 7.30 | 9.06 | -5.35 | 5.63 | **6.69** | **-15.19** | **14.48** | **14.00** |
| Bedtime | -7.22 | 7.81 | 10.17 | -4.4 | 5.05 | 6.52 | **37.09** | **38.15** | **36.75** |
| CAR | **19.78** | **20.71** | **24.07** | **35.78** | **35.89** | **38.05** | -8.25 | 8.15 | 7.87 |
| Slope | 10.49 | 10.67 | **13.43** | -2.3 | 2.44 | 2.90 | -11.94 | 11.68 | 10.61 |
| Urinary | 11.77 | 11.31 | 11.69 | -4.72 | 4.24 | 3.40 | **-12.33** | **12.58** | 9.06 |
| Resting | **-17.87** | **17.00** | 1.12 | **13.39** | **12.34** | 2.31 | 3.72 | 3.47 | **11.82** |
| AUC_g_ | **-25.55** | **25.19** | **30.47** | **34.06** | **34.42** | **40.12** | -11.5 | 11.48 | 9.90 |
| Total R^2^ | 0.13 | 0.13 | 0.11 | 0.04 | 0.03 | 0.03 | 0.03 | 0.03 | 0.03 |
|  | **Education** |  |  | **Personal growth** | |  | **CES-D** |  |  |
|  | Original | Sample | Sample/Rest | Original | Sample | Sample/Rest | Original | Sample | Sample/Rest |
| Waking | **35.06** | **33.95** | **35.06** | **18.77** | **19.13** | **16.86** | **-27.44** | **26.51** | **27.52** |
| Bedtime | **-27.09** | **28.27** | **28.15** | -4.58 | 5.80 | 5.48 | **32.05** | **33.49** | **36.18** |
| CAR | -10 | 9.75 | 9.91 | **50.45** | **50.96** | **41.62** | -4.43 | 4.30 | 4.59 |
| Slope | **15.55** | **15.16** | **15.53** | **10.87** | **10.88** | 9.31 | **-13.55** | **13.17** | **13.43** |
| Urinary | -0.99 | 1.18 | 1.18 | 5.48 | 4.12 | 2.21 | -10.23 | 10.57 | 9.95 |
| Resting | 1.69 | 2.21 | 0.56 | -0.83 | 0.45 | **16.95** | 4.55 | 4.24 | 0.29 |
| AUC_g_ | -9.61 | 9.47 | 9.61 | -9.02 | 8.65 | 7.57 | 7.76 | 7.73 | 8.05 |
| Total R^2^ | 0.04 | 0.04 | 0.04 | 0.02 | 0.02 | 0.03 | 0.04 | 0.03 | 0.03 |
|  | **BMI** |  |  | **Relationships** | |  | **Life satisfaction** | |  |
|  | Original | Sample | Sample/Rest | Original | Sample | Sample/Rest | Original | Sample | Sample/Rest |
| Waking | **-23.69** | **26.25** | **26.13** | **22.9** | **23.11** | **24.17** | **32.12** | **31.93** | **31.68** |
| Bedtime | -2.27 | 4.11 | 3.56 | -1.91 | 2.32 | 2.21 | -3.16 | 3.59 | 3.59 |
| CAR | -3.01 | 2.15 | 2.22 | **45.11** | **44.83** | **45.64** | 12.81 | 13.90 | 13.81 |
| Slope | **22.95** | **23.98** | **24.66** | **13.4** | **13.30** | **14.09** | **23.21** | **22.64** | **21.93** |
| Urinary | **-38.45** | **32.90** | **35.27** | 4.72 | 3.99 | 4.17 | 6.94 | 5.69 | 4.49 |
| Resting | -1.36 | 3.60 | 1.56 | 3.53 | 3.84 | 0.75 | -1.4 | 0.90 | 4.69 |
| AUC_g_ | 8.27 | 7.01 | 6.60 | -8.42 | 8.62 | 8.98 | **20.36** | **21.35** | **19.81** |
| Total R^2^ | 0.05 | 0.05 | 0.05 | 0.02 | 0.02 | 0.02 | 0.03 | 0.03 | 0.03 |
|  | **Chronic conditions** | |  | **Self-acceptance** | |  | **MASQ positive affect** | |  |
|  | Original | Sample | Sample/Rest | Original | Sample | Sample/Rest | Original | Sample | Sample/Rest |
| Waking | **-10.09** | **10.66** | **11.27** | **18.41** | **18.46** | **17.74** | **38.27** | **37.73** | **38.41** |
| Bedtime | 4.68 | 6.30 | 7.16 | -7.03 | 7.62 | 7.28 | -4.17 | 4.33 | 4.83 |
| CAR | -2.2 | 1.88 | 1.87 | **36.23** | **36.11** | **33.22** | **18.48** | **18.86** | **19.37** |
| Slope | -5.87 | 6.15 | 6.25 | 13.59 | 13.30 | 12.48 | **22.86** | **22.83** | **22.84** |
| Urinary | **-62.74** | **62.07** | **58.82** | 3.11 | 2.62 | 1.79 | 6.98 | 7.27 | 6.55 |
| Resting | 3.41 | 2.45 | 3.24 | -0.37 | 0.45 | 8.64 | -2.21 | 1.97 | 0.74 |
| AUC_g_ | **11.01** | **10.50** | **11.39** | **21.27** | **21.42** | **18.85** | -7.03 | 7.01 | 7.26 |
| Total R^2^ | 0.07 | 0.06 | 0.06 | 0.04 | 0.04 | 0.04 | 0.02 | 0.02 | 0.02 |
|  | **Perceived stress scale** | | | **Purpose in life** | |  | **MASQ anxious arousal** | |  |
|  | Original | Sample | Sample/Rest | Original | Sample | Sample/Rest | Original | Sample | Sample/Rest |
| Waking | **-23.71** | **22.91** | **21.76** | 7.28 | 8.55 | 6.88 | **-27.91** | **27.46** | **25.20** |
| Bedtime | 10.62 | 10.97 | 10.23 | **-53.99** | **53.92** | **37.61** | **21.95** | **22.32** | **20.19** |
| CAR | **-26.61** | **26.81** | **24.67** | **11.66** | **13.06** | **9.56** | -3.01 | 2.97 | 2.78 |
| Slope | **29.16** | **29.16** | **26.79** | 8.62 | **8.70** | 6.18 | -11.89 | 11.71 | 10.76 |
| Urinary | -2.58 | 2.61 | 1.84 | **9.27** | 6.88 | 2.90 | **-22.92** | **23.25** | **17.26** |
| Resting | -0.77 | 0.99 | 8.46 | -0.86 | 0.64 | **30.70** | 0.4 | 0.41 | 13.84 |
| AUC_g_ | 6.54 | 6.55 | 6.25 | -8.32 | 8.25 | 6.17 | -11.91 | 11.88 | 9.96 |
| Total R^2^ | 0.01 | 0.01 | 0.01 | 0.02 | 0.02 | 0.03 | 0.07 | 0.07 | 0.08 |
|  | **Difficulty paying bills** | | | **Environmental** | |  | **MASQ anxious symptoms** | |  |
|  | Original | Sample | Sample/Rest | Original | Sample | Sample/Rest | Original | Sample | Sample/Rest |
| Waking | **-18.54** | **18.93** | **19.51** | 10.47 | 10.49 | 10.94 | **-18.87** | **17.52** | 15.96 |
| Bedtime | 6.76 | 9.29 | 10.03 | -8.74 | 10.30 | 10.14 | 3.99 | 4.03 | 3.24 |
| CAR | **-23.35** | **25.14** | **25.98** | **-11.93** | **12.80** | **12.87** | **-20.06** | **20.14** | **17.31** |
| Slope | 14.49 | 13.57 | 13.66 | 3.89 | 3.92 | 4.12 | 13.1 | 12.60 | 11.26 |
| Urinary | -7.2 | 4.19 | 3.65 | **26.33** | **22.16** | **21.55** | -13.15 | 13.68 | 9.59 |
| Resting | 4.48 | 2.25 | 0.82 | 2.18 | 3.31 | 3.22 | -2.23 | 2.94 | **19.25** |
| AUC_g_ | **-25.18** | **26.62** | **26.34** | **36.46** | **37.02** | **37.17** | **-28.59** | **29.08** | **23.41** |
| Total R^2^ | 0.02 | 0.02 | 0.02 | 0.01 | 0.01 | 0.01 | 0.01 | 0.01 | 0.02 |

Original = unadjusted; Adj (sample) = adjusted for time between Main, Diary, and Biomarker samples; Adj (sample/rest) = additional adjustment for time of resting sample collection. Negative values represent negative regression coefficients.
